# Supplementary material for: Practice Recommendations for the Management of MASLD in Primary Care: Consensus Results
Source: Diseases. 2024 Aug 10;12(8):180. doi: 10.3390/diseases12080180 (PMC11353634; doi:10.3390/diseases12080180)
Supplement: Supplementary file 1 [file diseases-12-00180-s001.zip › diseases-3085532-supplementary.pdf]

Supplemental material 1 – Round one statements and questionnaire

We thank you for your contribution to the upcoming consensus meeting. In preparation for the meeting, we ask for your participation in a first-round Delphi panel exercise based on the RAND/UCLA Modified Panel Method (Broder et al 2022, https://www.ncbi.nlm.nih.gov/pmc/articles/PMC9130741/. We will have the opportunity to discuss the findings of this survey in the consensus meeting where the second-round survey will be completed.

Instructions for Delphi exercise
Below you will find a list of recommendations for the management of NAFLD in Primary Care. We kindly ask you to rank your level of agreement by marking an 'X' in the appropriate place. For additional comments/suggestions for improved wording please use space provided. If you can submit your responses in advance of the consensus meeting to allow our team to conduct a rapid analysis of the first-round Delphi exercise.

A) RISK SCREENING AND DIAGNOSIS

R1: General practitioners (*GPs) and other primary health care (PHC) professionals should consider persons with indications of metabolic dysfunction, including overweight or obesity, type two diabetes, determinants of metabolic syndrome and/or persistently elevated aminotrasferase levels as 'high risk' for developing NAFLD and NASH.*

Level of agreement:	1: Strong disagreement	2: High disagreement	3: Neutral	4: High agreement	5: Strong agreement

Comments:

R2: *GPs and other PHC professionals should use prediction calculators to assess liver fibrosis and, particularly, the FIB-4 index which has been shown to be easily accepted and implemented in primary care settings.*

Level of agreement:	1: Strong disagreement	2: High disagreement	3: Neutral	4: High agreement	5: Strong agreement

Comments:

R3: *Transient elastography is recommended as the appropriate imaging technique to assess and stage the degree of fibrosis in people with NAFLD.*

Level of agreement:	1: Strong disagreement	2: High disagreement	3: Neutral	4: High agreement	5: Strong agreement

Comments:

R4: *GPs and other PHC professionals should use prediction calculations to assess cardiovascular (CVD) risk, including the CVD score by the European Society of Cardiology.*

Level of agreement:	1: Strong disagreement	2: High disagreement	3: Neutral	4: High agreement	5: Strong agreement

Comments:

PATIENT'S HEALTH MONITORING

R5: *Persons with a mild or moderate risk for advanced liver disease should be assessed with FIB-4 test every 6 months.*

Level of agreement:	1: Strong disagreement	2: High disagreement	3: Neutral	4: High agreement	5: Strong agreement

Comments:

B) REFERRAL TO SPECIALISTS

R6: *Persons with a high risk for advance disease (FIB-4≥2.67 and/or transient elastography, as above 7.9 kPa) should be referred to a specialist for further assessment and treatment.*

Level of agreement:	1: Strong disagreement	2: High disagreement	3: Neutral	4: High agreement	5: Strong agreement

Comments:

R7: *Persons with a high risk for advance disease (FIB-4≥2.67 and/or transient elastography, as above 7.9 kPa) should be supported for weight reduction and smoking cessation in primary care and referred to specialized services, if needed.*

Level of agreement:	1: Strong disagreement	2: High disagreement	3: Neutral	4: High agreement	5: Strong agreement

Comments:

C) MANAGEMENT OF NAFLD
LIFESTYLE INTERVENTIONS

R8: GPs and other PHC professional should offer interventions including weight loss, smoking cessation and restrictions in alcohol use for the management of NAFLD/NASH.

Level of agreement:	1: Strong disagreement	2: High disagreement	3: Neutral	4: High agreement	5: Strong agreement

Comments:

R9: *GPs and other PHC professionals should use very brief advice and motivational interviewing interventions for lifestyle change in every consultation with a patient with high risk or confirmed diagnosis of NAFLD/NASH.*

Level of agreement:	1: Strong disagreement	2: High disagreement	3: Neutral	4: High agreement	5: Strong agreement

Comments:

R10: *GPs and other PHC professionals should promote NAFLD/NASH awareness and health literacy among all of their patients.*

Level of agreement:	1: Strong disagreement	2: High disagreement	3: Neutral	4: High agreement	5: Strong agreement

Comments:

D) PHARMACOLOGICAL TREATMENT FOR NAFLD/NASH

R11: In patients with NAFLD and biopsy proven NASH and type two diabetes, GPs and other PHC professionals should consider treatment with GLP-1 RAs and pioglitazone.

Level of agreement:	1: Strong disagreement	2: High disagreement	3: Neutral	4: High agreement	5: Strong agreement

Comments:

E) PHARMACOLOGICAL TREATMENT FOR CO-MORBIDITY

R12: *To reduce the cardiovascular risk in patients with NAFLD/NASH, GPs and other PHC professionals should consider treatment with GLP-1 RAs, pioglitazone or SGLT2 inhibitors.*

Level of agreement:	1: Strong disagreement	2: High disagreement	3: Neutral	4: High agreement	5: Strong agreement

Comments:

R13: *GPs and other PHC professionals should consider seraglutide 2.4mg/week or liraglutide 3mg/day as a treatment option for persons with NAFLD or NASH and a BMI>=27kg/m2 as an adjunctive therapy to promote lifestyle modification and improve cardiovascular risk.*

Level of agreement:	1: Strong disagreement	2: High disagreement	3: Neutral	4: High agreement	5: Strong agreement

Comments:

F) SURGICAL MANAGEMENT

R14: *GPs and other PHC professionals should consider bariatric surgery as a therapy and improvement of the cardiovascular risk in persons with NAFLD and a BMI of 35 kg/m2 (in European population) and refer them to a specialist for a final decision.*

Level of agreement:	1: Strong disagreement	2: High disagreement	3: Neutral	4: High agreement	5: Strong agreement

Comments:

G) INTEGRATED CARE

R15: *GPs and other PHC professionals should collaborate with laboratory personnel, specialists to promote health and well-being of patients with NAFLD.*

Level of agreement:	1: Strong disagreement	2: High disagreement	3: Neutral	4: High agreement	5: Strong agreement

Comments:
